# Interventions to Address Cardiovascular Risk in Obese Patients: Many Hands Make Light Work

**DOI:** 10.3390/jcdd10080327

**Published:** 2023-07-31

**Authors:** Valeria Visco, Carmine Izzo, Davide Bonadies, Federica Di Feo, Giuseppe Caliendo, Francesco Loria, Costantino Mancusi, Pierpaolo Chivasso, Paola Di Pietro, Nicola Virtuoso, Albino Carrizzo, Carmine Vecchione, Michele Ciccarelli

**Affiliations:** 1 Department of Medicine, Surgery and Dentistry, University of Salerno, 84081 Baronissi, Italy; vvisco@unisa.it (V.V.); carmine.izzo93@gmail.com (C.I.); d.bonadies1@gmail.com (D.B.); fed.difeo@outlook.it (F.D.F.); giuseppecaliendo1995@gmail.com (G.C.); francescoloria94@gmail.com (F.L.); pdipietro@unisa.it (P.D.P.); acarrizzo@unisa.it (A.C.); cvecchione@unisa.it (C.V.); 2 Department of Advanced Biomedical Sciences, Federico II University of Naples, 80138 Naples, Italy; costantino.mancusi@unina.it; 3 Department of Emergency Cardiac Surgery, Cardio-Thoracic-Vascular, University Hospital “San Giovanni di Dio e Ruggi D’Aragona”, 84131 Salerno, Italy; pierpaolo.chivasso@sangiovannieruggi.it; 4 Cardiology Unit, University Hospital “San Giovanni di Dio e Ruggi d’Aragona”, 84131 Salerno, Italy; n1virtuoso@gmail.com; 5 Vascular Physiopathology Unit, IRCCS Neuromed, 86077 Pozzilli, Italy

**Keywords:** obesity, cardiovascular risk, cardiometabolic complications, lifestyle interventions, diet, physical activity, pharmacologic treatment, bariatric surgery

## Abstract

Obesity is a growing public health epidemic worldwide and is implicated in slowing improved life expectancy and increasing cardiovascular (CV) risk; indeed, several obesity-related mechanisms drive structural, functional, humoral, and hemodynamic heart alterations. On the other hand, obesity may indirectly cause CV disease, mediated through different obesity-associated comorbidities. Diet and physical activity are key points in preventing CV disease and reducing CV risk; however, these strategies alone are not always sufficient, so other approaches, such as pharmacological treatments and bariatric surgery, must support them. Moreover, these strategies are associated with improved CV risk factors and effectively reduce the incidence of death and CV events such as myocardial infarction and stroke; consequently, an individualized care plan with a multidisciplinary approach is recommended. More precisely, this review explores several interventions (diet, physical activity, pharmacological and surgical treatments) to address CV risk in obese patients and emphasizes the importance of adherence to treatments.

## 1. Introduction: Relationship between Obesity and Cardiovascular Disease

Obesity is defined when the body mass index (BMI) exceeds a value of 30 kg/m^2^ [1]. This condition is a growing public health epidemic across the world. Accordingly, the available data suggest that the global burden of obesity has more than tripled since 1975 [2], not only in first-world countries; indeed, recent evidence shows that obesity is spreading in low-and middle-income countries [3]. Specifically, around 42% of adults in the US are obese, while prevalence has increased to about 25% in the United Kingdom [4]. Italy follows this trend with an increase of almost 30% in the adult obese population in the last three decades [5].

Obesity is a multifactorial condition influenced by a range of social and environmental factors. While individual behaviors, such as diet and physical activity, play a role in obesity development, broader determinants related to society and the environment also significantly contribute.

The social and environmental determinants of obesity include various aspects.

First, lower socioeconomic status is associated with a higher prevalence of obesity. Factors such as limited access to healthy food options, higher levels of food insecurity, and reduced opportunities for physical activity contribute to the higher obesity rates observed in disadvantaged populations. Socioeconomic disparities can influence dietary choices, availability of resources, and overall health-related behaviors [6].

Another important factor includes the food environment, which consists of the availability and accessibility to healthy food options in communities. Areas characterized by a high density of fast-food outlets, limited access to grocery stores offering fresh produce, and a lack of affordable healthy food options are associated with higher obesity rates. These “food deserts” make it challenging for individuals to make nutritious choices, leading to a higher consumption of energy-dense, nutrient-poor foods [7,8].

Cultural norms and social norms influence and play a role in shaping dietary preferences and behaviors. Societal norms that prioritize large portion sizes, frequent consumption of high-calorie foods, and sedentary lifestyles can contribute to the development of obesity. Additionally, social networks and peer influence can impact food choices and physical activity patterns [9].

Limited education and low health literacy levels can hinder individuals’ understanding of nutrition and the importance of a healthy lifestyle. Insufficient knowledge about healthy food choices, portion sizes, and the benefits of regular physical activity can contribute to obesity. Educational interventions and improving health literacy can empower individuals to make informed decisions and adopt healthier behaviors [10].

Environmental determinants of obesity include the built environment, such as the design and layout of neighborhoods and urban spaces that can affect physical activity levels. Accessible sidewalks, bike lanes, parks, and recreational facilities can promote active lifestyles. Conversely, neighborhoods lacking these features, characterized by limited safety and opportunities for physical activity, can discourage exercise and contribute to sedentary behaviors [11].

Moreover, transportation infrastructure in terms of availability and quality of public transportation systems influence physical activity levels. Communities with inadequate public transportation options may rely more on sedentary modes of transportation, such as cars, leading to reduced opportunities for physical activity [12].

In modern society, advertising and marketing and, in particular, aggressive marketing of unhealthy foods, especially towards children, can contribute to poor dietary choices and increased consumption of energy-dense foods. Exposure to advertisements promoting sugary beverages, fast food, and unhealthy snacks can influence eating behaviors and contribute to obesity [13].

Finally, the work environment, including occupational factors, such as sedentary work environments and long working hours, can limit opportunities for physical activity and contribute to a sedentary lifestyle. Workplace policies and environments that promote physical activity, such as standing desks or workplace wellness programs, can help mitigate the impact of sedentary jobs [14].

Obesity is implicated in recent slowing in improved life expectancy [15] and increases the risk of several chronic diseases (diabetes, hypertension, coronary heart disease) [16,17,18,19]. On average, in the populations studied (largely Caucasians), obesity class III (BMI ≥ 40 kg/m^2^) shortens life expectancy by approximately 10 years, and obesity class I (BMI 30–34.9 kg/m^2^) reduces life duration by around 3 years, relative to normal weight, with the number of years lost varying according to age, sex, and severity of obesity [1,20,21]. Specifically, the metabolic syndrome, a concurrence of disturbed glucose and insulin metabolism, overweight and abdominal fat distribution, mild dyslipidemia, and hypertension, is most important because of its association with cardiovascular disease (CVD) [22,23]. More precisely, CV risk is high/very high with obesity class I, very high with class II, and extremely high with class III in Caucasian populations [1]. However, BMI indicates the overall excess body weight, while waist circumference better defines fat distribution and abdominal body fat [24,25]. Additionally, BMI is inadequate for defining body composition and the difference between fat mass and fat-free mass; specifically, the CV risk differs according to the type of ectopic deposition [1,24,26,27]. Specifically, the distribution of adipose tissue has clinical importance, because central adiposity, especially visceral obesity, is more deleterious, while lower body fat deposition may be actually protective [28]. However, the implementation of fat distribution assessment in clinical practice remains a challenge: the instruments used have a high acquisition cost and specialized professionals are needed to use them [29].

**Figure 1:**
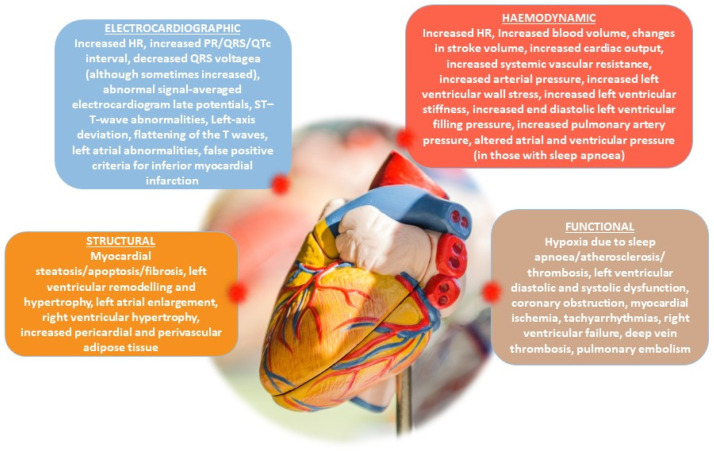
The key electrocardiographic, hemodynamic, structural and functional changes associated with obesity.

Genetic and population-based cohort analysis has revealed a direct relationship between adiposity and other high-risk CV traits, including aortic diseases, HF, deep vein thrombosis, hypertensive heart disease, peripheral artery diseases, and atrial fibrillation (AF) [26,30]. Indeed, several obesity-related mechanisms drive structural, functional, humoral, and hemodynamic alterations believed to underpin the development of CVD (Figure 1) [1]. Specifically, there is a causal association between adipose tissue and CVD: obesity is the causal pathway of several traditional CV risk factors such as atherogenic dyslipidemia, hypertension and diabetes; moreover, obesity-related OSA contributes to CVD risk with the promotion of hypoxia, cardiac dysrhythmias, insulin resistance, and hypertension [31,32,33,34]. Obesity often leads to a deterioration of adipose tissue plasticity, which is linked to fibrosis, inflammation, progenitor cell senescence, and catecholamine resistance [35]. On the other hand, obesity may indirectly cause CVD, mediated through different obesity-associated comorbidities: an excessive increase in body weight prejudices mobility and physical activity and/or worsens musculoskeletal comorbidities and subsequently reduces energy expenditure, resulting in a vicious cycle of weight gain and rising CV risk [36,37,38]; moreover, total blood volume and cardiac output are higher in people with overweight/obesity, contributing to structural and functional changes to the heart and vascular system (left ventricular hypertrophy (LVH), left ventricular diastolic dysfunction and predisposition to HF [37,38,39,40]).

These obesity-associated changes in cardiac function have been described as the “cardiomyopathy of obesity” [41]. Sensitive measures of contractile function, such as left ventricular fractional shortening, systolic velocity, and myocardial strain (circumferential and longitudinal), are impaired in the setting of obesity and/or metabolic syndrome [42,43,44,45]. Notably, cardiac functional responses to physiologic perturbations (e.g., exercise), pathologic conditions (e.g., myocardial ischemia) [46,47,48], or pharmacologic stimuli (e.g., catecholamines, glucagon-like-peptide-1 mimetics) [49] are also known to be significantly influenced by an obese/metabolic syndrome phenotype. Investigators in this field have identified relevant and important molecular pathways linking obesity to CV continuum and cardiac dysfunction; in particular, alterations in myocardial Ca2+ handling via changes in the functional expression of SERCA2A and ryanodine (RyR2) receptors [50,51] have been of interest. Also of note is a growing interest in modifications in the regulation of myocardial titin (which influences the passive and restoring force of the cardiac sarcomere and can contribute to hypertrophic signaling) as a potential target or mediator of obesity-associated cardiac dysfunction [50,52,53]. Moreover, it has been reported that weight loss through lifestyle interventions in obese patients with heart failure may result in improvements in the New York Heart Association classification, quality of life and exercise capacity [54].

One added possibility is that progressive vascular disease further influences these heart changes through mechanisms specific to the atherosclerotic process or microvascular dysfunction, independent of the obese state [55,56,57].

There is a growing recognition of the importance of population-level interventions, and individualized care plans with a multidisciplinary approach are recommended [25,58,59]; indeed, the multidisciplinary interventions describe major changes in body composition, and the recurring pattern in clinical trials is an energy reduction and control in the percentage of intake of macronutrients along with the performance of regularly structured exercise [60].

Consequently, this study explores several interventions (diet, physical activity, pharmacological and surgical treatments) to address CV risk in obese patients. These interventions are necessary to improve CV risk profile and mortality and prevent CV events. Specifically, weight loss beneficially affects traditional CVD risk factors (e.g., hypertension, atherogenic dyslipidemia, and T2DM), but relapse is common without long-term support [61]; moreover, a weight threshold needs to be reached before mortality benefit is achieved [1]. Consequently, the hypothesis is that some treatments and interventions can be superior and more lasting over time, especially if combined with each other.

## 2. Methods

We conducted a detailed search on interventions to address CV risk in people with obesity: diet, physical activity, pharmacological and surgical treatments. Articles reviewed in this paper were searched in journal databases such as PubMed and subject-specific professional websites, including Google Scholar. The search terms included: obesity; overweight; CV risk; cardiometabolic complications; CV disease; CV mortality; metabolic syndrome; adiposity; lifestyle interventions; diet; physical activity; pharmacologic treatment; bariatric surgery. Inclusion criteria focus on those articles directly or indirectly related to the topic of obesity and CV risk.

Both qualitative and quantitative reports were reviewed. Qualitative reports provide insights into the problem by helping researchers understand reasons, opinions, and motivations. On the other hand, quantitative articles use measurable data to formulate facts and discover patterns in research. The authors independently screened titles and abstracts. Full texts were sourced for relevant articles. The included trials and meta-analyses reference lists were also reviewed for relevant articles. However, our article is mainly a narrative review since we did not follow a standardized protocol, such as that of systematic reviews.

## 3. Lifestyle Modifications: Diet and Physical Activity

### 3.1. Diet

As mentioned, overweight and obesity are strongly associated with comorbidities such as hypertension and insulin resistance and contribute to developing CV diseases and the resultant morbidity and mortality. The cornerstone in the treatment of obesity is diet, but can diet influence the CV risk in these patients? More precisely, fat distribution is essential: individuals with higher visceral adipose tissue and ectopic fat deposition have an increased prevalence of cardiometabolic disorders, including hypertension, dyslipidemia, and insulin resistance [62]. Among different mechanisms, lipid metabolism is mostly influenced by ectopic fat depots; indeed, intracellular accumulation of nonesterified fatty acids and triglycerides promotes endoplasmic reticulum stress, mitochondrial uncoupling, oxidative stress, and altered membrane composition/function, promoting inflammatory response and cell death with local and systemic effects [63,64].

Moreover, many genes affect the response to diets, opening the possibility of “personalized medicine” for managing obesity [65]. Specifically, with sequencing and analytical approaches, the developments in precision nutrition have the potential to lead to new paradigms, which can further lead to customized treatments specific to individuals or susceptible groups of individuals [66,67].

In tailoring diet programs, personal preferences should be considered to maximize compliance with hypocaloric diet prescriptions. Moreover, concerning specific diet recommendations, a hypocaloric diet with less than 1200 Kcal/day may increase the risk of micronutrient deficiencies and have a low probability of providing long-lasting weight loss [25]; an appropriate approach is to prescribe a 15–30% reduction of caloric intake from habitual energy intake, to increase patient adherence [68].

**Figure 2:**
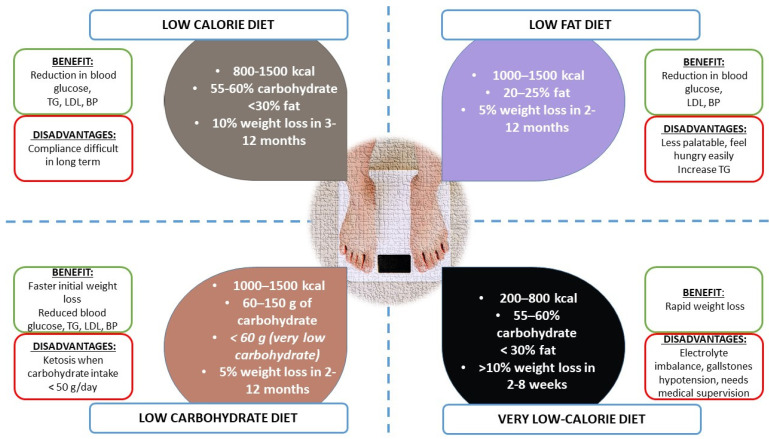
Comparison of different dietary regimes.

There is evidence that diet can promote the control of CV risk factors, such as hypertension, as demonstrated in the DASH (Dietary Approaches to Stop Hypertension) trial; a diet that emphasizes fruits, vegetables, and low-fat dairy foods and that has reduced amounts of total and saturated fat and cholesterol lowers blood pressure substantially both in people with hypertension and those without hypertension [69]. Most of the studies are of a specific type of diet and how it impacts weight and CV risk. In general, there are four types of dietary regimens used in the treatment of overweight or obese persons:

1. Low-calorie diet (LCD)
2. Low-fat diet
3. Low-carbohydrate diet (e.g., Mediterranean Diet)
4. Very low-calorie diet (VLCD) [70] (Figure 2).

The “PREvencion con DIeta MEDiterranea (PREDIMED) intervention trial” is one of the main trials that support the effectiveness of the Mediterranean Diet for CV prevention [71]. There is also evidence that it is associated with lower inflammation and reduced risk of diabetes and CVD [72,73]. The CARDIVEG Study (Cardiovascular Prevention With Vegetarian Diet) compares the vegetarian diet and Mediterranean diet: both effectively reduced body weight, BMI, and fat mass, with no significant differences. However, the vegetarian diet was more effective in reducing low-density lipoprotein cholesterol levels, whereas the Mediterranean diet led to a more significant reduction in triglyceride levels [74]. A ketogenic diet, a high-fat, low-carbohydrate, and adequate-protein diet, is another strategy for weight loss in obese people. It is associated with a beneficial effect on CV risk, and several mechanisms are proposed, such as reducing inflammation and increasing mitochondrial biogenesis [75]. It is commonly used for short periods because of the restrictive regimen, so there is little evidence of long-term effects [76]. A 2022 study demonstrates differential effects between a Very Low Carbohydrate Ketogenic Diet (VLCKD) versus standard treatment with low carbohydrate plus bariatric surgery on remodeling the inflammatory and oxidative status of patients with obesity [77]. Results support the immunomodulatory effect of nutritional ketosis induced by a VLCKD synergistically with weight loss as a strategy to improve innate immunity and prevent infections and carcinogenesis in patients with obesity [77].

Moreover, some most recent studies indicate that dietary habits and obesity seem to be influenced also by chronotype (individual’s preference for the timing of sleeping, eating, and activity in a day) [78]. More precisely, late types usually present unhealthy eating behaviors (eating late at night, skipping breakfast, and eating processed foods), while early types are more likely to have healthy habits (eating early and predominantly fresh/minimally processed foods); accordingly, late types frequently present higher weight and BMI [78]. Accordingly, a diet adjusted to the chronotype has been seen to be more effective than conventional hypocaloric diet therapy in terms of anthropometric parameters. Additionally, it has been shown that bariatric surgery is less effective in weight loss in evening chronotype than in morning chronotype patients, and the evening chronotypes are less successful in long-term weight control [79].

The diet–drug association is also to be considered in approaching obesity treatment. The European Guidelines for obesity management consider drugs in patients with BMI > 30 or BMI > 27 and comorbidities [80]. In particular, in patients with comorbidities such as metabolic syndrome or diabetes, drugs can help to control this additional risk factor, although it is not possible to conclude that there is an optimal long-term diet–drug combination for procuring weight loss or weight maintenance for anyone. Furthermore, due to the possible adverse effects of the prescribed drugs, caution is warranted [81].

### 3.2. Physical Activity

Physical activity is any bodily movement produced by skeletal muscles that require energy expenditure, while exercise is a planned, structured, and repetitive subset of physical activity aimed at maintaining or improving one or more components of physical fitness [82]. In obesity management, various exercise interventions have been studied to determine their effectiveness in promoting weight loss and improving health outcomes.

Specifically, physical activity plays a crucial role in managing obesity, promoting weight loss, and improving health outcomes. The following sections provide an in-depth exploration of the various exercise interventions commonly employed for obese patients, discussing their effectiveness and potential benefits. The chapter covers (1) aerobic exercise, (2) resistance exercise, (3) combined aerobic and resistance exercise, and (4) high-intensity interval training (HIIT).

#### 3.2.1. Type of Exercise

##### Aerobic Exercise

Aerobic exercise, or cardio, involves activities that increase heart rate and respiration, utilizing large muscle groups and improving oxygen consumption. Some examples include walking, jogging, cycling, and swimming. Aerobic exercise has been consistently demonstrated to improve CV fitness, reduce body fat, and improve metabolic health in obese individuals [83].

Aerobic exercise is particularly effective in improving CV health in obese patients, as it enhances CV fitness, reduces blood pressure, and improves blood lipid profiles. For example, a meta-analysis by Kelley et al. [84] showed that aerobic exercise significantly reduced systolic and diastolic blood pressure in obese individuals, reducing the risk of hypertension and associated health complications.

Aerobic exercise can also improve metabolic health, as it has been shown to increase insulin sensitivity and glucose tolerance in obese individuals [85]. These improvements in metabolic health can help reduce the risk of developing type 2 diabetes and improve glycemic control in those already diagnosed with the condition.

Aerobic exercise promotes weight loss and reduces body fat in obese individuals. A systematic review by Wu et al. [86] demonstrated that aerobic exercise significantly reduced body weight, BMI, and waist circumference. Significantly, aerobic exercise can also help preserve lean body mass during weight loss, which is crucial for maintaining metabolic health and preventing weight regain.

##### Resistance Exercise

Resistance exercise, or strength training, involves activities that require muscular effort to overcome external resistance, such as lifting weights or using resistance bands. Resistance exercise has increased lean body mass, reduced fat mass, and improved insulin sensitivity in obese patients [87].

Resistance exercise is particularly beneficial for improving musculoskeletal health in obese individuals. It has been shown to increase muscle strength, power, and endurance, as well as improve bone mineral density and reduce the risk of osteoporosis [88]. These musculoskeletal benefits are crucial for maintaining functional independence and preventing injuries in obese patients.

Similar to aerobic exercise, resistance exercise has been shown to improve insulin sensitivity and glucose tolerance in obese individuals [87]. Additionally, increasing lean body mass through resistance training increases resting metabolic rate, which may help promote weight loss and weight maintenance over time [89].

##### Combined Aerobic and Resistance Exercise

Several studies have suggested that combining aerobic and resistance exercise may reduce body weight, waist circumference, and body fat percentage more effectively than aerobic exercise alone [90]. Moreover, this combined approach may provide synergistic benefits, optimizing body composition and improving overall health outcomes in obese individuals [91].

A meta-analysis by Clark [92] found that combined aerobic and resistance exercise led to more important reductions in body weight and fat mass compared to aerobic or resistance exercise alone. The combination of exercise modalities may help target different body composition and metabolism aspects, leading to enhanced weight loss and improved overall health.

Combined aerobic and resistance exercise may offer additional metabolic benefits, such as improved insulin sensitivity, glucose tolerance, and lipid profiles, compared to either modality alone [91]. By incorporating both types of exercise, individuals can reap the distinct advantages of each while maximizing overall metabolic health improvements.

Incorporating aerobic and resistance exercise can also improve functional capacity, balance, and mobility in obese individuals [93]. Improved functional capacity is crucial for maintaining independence, preventing falls, and enhancing the overall quality of life.

HIIT is a form of exercise characterized by short bursts of high-intensity activity interspersed with rest periods or low-intensity exercise. HIIT has gained popularity recently due to its time-efficient nature and potential to elicit substantial health benefits in a shorter time than traditional continuous aerobic exercise [94].

HIIT has been shown to improve CV fitness, insulin sensitivity, and glucose tolerance in obese individuals, potentially even more effectively than continuous aerobic exercise [95]. A meta-analysis by Wewege et al. [96] found that HIIT led to similar improvements in cardiorespiratory fitness as moderate-intensity continuous training, despite a shorter total exercise time.

Although the evidence is less consistent than it is for aerobic exercise, several studies have demonstrated that HIIT can reduce body weight, body fat, and waist circumference in obese individuals [97]. Moreover, the time-efficient nature of HIIT may make it a more accessible and sustainable exercise option for some individuals, potentially improving adherence and long-term weight loss outcomes.

It is essential to consider the increased risk of injury and the need for proper supervision when implementing HIIT in obese individuals [94]. Furthermore, as obese individuals may have reduced exercise capacity and increased musculoskeletal stress, it is crucial to gradually progress the intensity and duration of HIIT sessions, as well as to ensure proper technique to minimize injury risk.

Aerobic exercise, resistance exercise, combined aerobic and resistance exercise, and high-intensity interval training have all been shown to provide significant health benefits for obese individuals. Each exercise modality offers unique advantages, and a combination of these modalities may be optimal for promoting weight loss, improving CV and metabolic health, and enhancing the overall quality of life in obese patients. The choice of exercise intervention should consider the individual’s preferences, goals, and current fitness levels to maximize adherence and long-term success in obesity management.

#### 3.2.2. Exercise Duration and Frequency

The American College of Sports Medicine (ACSM) advises a minimum of 150 min of moderate-intensity aerobic exercise or 75 min of vigorous-intensity aerobic exercise per week, along with two or more days of muscle-strengthening activities for general health benefits [98]. This chapter provides an in-depth analysis of the impact of exercise duration and frequency on weight loss and health outcomes in obese individuals, addressing the following topics: (1) the role of moderate- and vigorous-intensity exercise, (2) dose-response relationship between exercise and health outcomes, and (3) exercise adherence and strategies for maintaining regular exercise.

Moderate-intensity exercise refers to activities that raise heart rate and breathing rate but still allow for conversation, such as brisk walking, swimming, or cycling at a leisurely pace. In contrast, vigorous-intensity exercise involves activities that significantly increase heart and breathing rates, making it challenging to carry on a conversation, such as running, fast cycling, or participating in high-intensity fitness classes [98].

Jakicic et al. [99] discuss the role of physical activity and exercise in treating patients with overweight and obesity, emphasizing that adhering to the ACSM guidelines effectively reduces body weight and improves metabolic health in obese individuals. However, it is essential to note that increasing exercise duration and frequency may lead to even more significant health benefits.

#### 3.2.3. Dose–Response Relationship between Exercise and Health Outcomes

The dose–response relationship between exercise and health outcomes refers to the changes in health parameters as a function of the amount (dose) of exercise performed. A study by Donnelly et al. [100] explored this relationship, to determine whether increasing exercise duration and frequency beyond the ACSM’s guidelines would lead to additional health benefits. The researchers found that obese individuals who exceeded the minimum exercise recommendations experienced more significant reductions in body weight, waist circumference, and body fat percentage, as well as improved insulin sensitivity, glucose tolerance, and lipid profiles. These findings suggest that a higher dose of exercise may confer more significant health benefits in obese individuals.

However, it is essential to consider the risk of overtraining and injury when increasing exercise duration and frequency, as excessive exercise can lead to adverse health outcomes and reduced adherence to exercise programs [101]. Consequently, it is crucial to balance optimizing health benefits and minimizing the risk of overtraining and injury.

#### 3.2.4. Exercise Adherence and Strategies for Maintaining Regular Exercise

**Figure 3:**
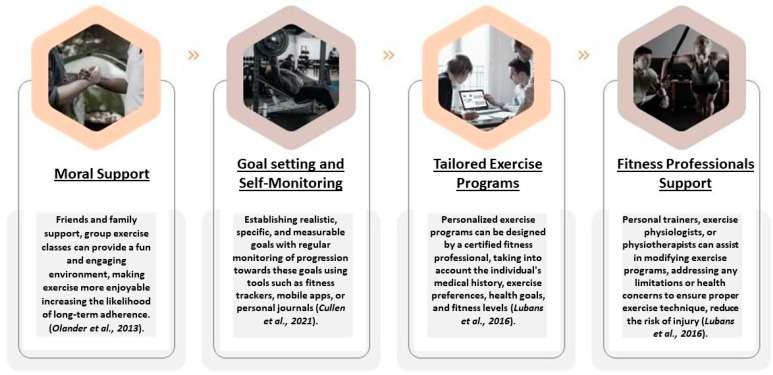
Strategy to enhance exercise adherence in obese patients [87,94,97,102].

Adherence to exercise programs is a significant factor in determining the long-term success of obesity management. A systematic review by Teixeira et al. [102] identified several key determinants of exercise adherence in obese individuals, including self-efficacy, intrinsic motivation, social support, and enjoyment of exercise. Additionally, factors such as accessibility, cost, and time constraints can influence adherence to exercise programs. Specifically, to enhance exercise adherence, several strategies have been proposed in the literature (Figure 3).

The ACSM’s recommended exercise guidelines provide a solid foundation for promoting weight loss and improving health outcomes in obese individuals. However, increasing exercise duration and frequency beyond these guidelines may lead to additional health benefits, provided that overtraining and injury risks are carefully managed. Adherence to exercise programs is crucial for long-term success in obesity management. Personalized exercise programs, group exercise, social support, goal setting and self-monitoring, overcoming barriers, and professional guidance can help improve exercise adherence and optimize health outcomes for obese individuals [103].

#### 3.2.5. Exercise and Mental Health in Obese Patients

**Figure 4:**
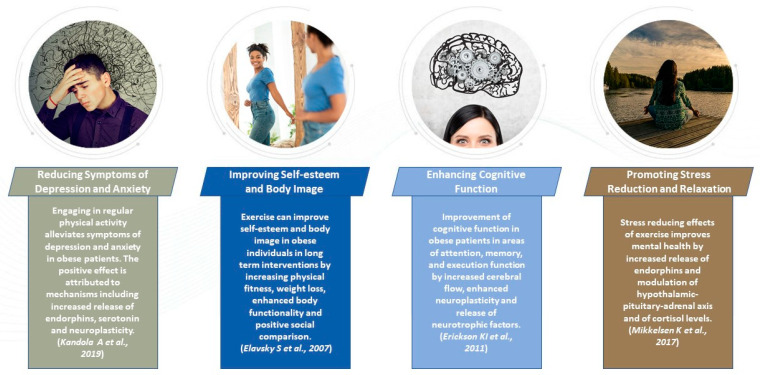
Impact of exercise on mental health in obese patients [102,104,105,106].

Physical activity not only contributes to weight loss and overall physical health but also plays a significant role in enhancing mental health in obese individuals. This chapter delves into how exercise can positively impact mental health, including: reducing symptoms of depression and anxiety, improving self-esteem and body image, enhancing cognitive function, and promoting stress reduction and relaxation (Figure 4).

Specifically, exercise is vital in promoting mental health in obese individuals. Regular physical activity can decrease symptoms of depression and anxiety, improve self-esteem and body image, enhance cognitive function, and promote stress reduction and relaxation. In conclusion, incorporating exercise into the overall treatment plan for obesity can lead to improved mental health outcomes and contribute to long-term weight management success.

#### 3.2.6. Exercise and Appetite Regulation in Obese Patients

In addition to its effects on physical and mental health, exercise can also regulate appetite in obese individuals. Specifically, this chapter examines how exercise can influence appetite regulation, including the impact on hunger and satiety hormones, exercise-induced changes in energy expenditure, and the role of exercise intensity and duration.

##### Impact on Hunger and Satiety Hormones

Exercise can influence the release of hunger and satiety hormones, crucial in regulating appetite. A study by Martins et al. [107] found that acute bouts of exercise led to changes in the release of appetite-regulating hormones such as ghrelin, peptide YY (PYY), and glucagon-like peptide-1 (GLP-1), which contributed to a decrease in appetite and energy intake in obese individuals.

Ghrelin is a hormone that stimulates hunger and food intake. Research has demonstrated that exercise can lead to a transient decrease in circulating ghrelin levels, contributing to a reduction in appetite [107]. However, the specific response to exercise may vary depending on the individual and the type, intensity, and duration.

PYY and GLP-1 are hormones that promote satiety and reduce appetite. Exercise has been shown to increase circulating levels of PYY and GLP-1 in both lean and obese individuals [108]. This increase in satiety hormones can help counteract the hunger-promoting effects of ghrelin, leading to a reduction in overall appetite.

##### Exercise-Induced Changes in Energy Expenditure

Exercise can also impact appetite regulation by increasing energy expenditure. By expending more energy through physical activity, obese individuals may experience a compensatory decrease in appetite to maintain energy balance [109]. However, it is essential to note that individual responses to exercise-induced changes in energy expenditure can vary and may not always lead to a reduction in appetite.

##### Role of Exercise Intensity and Duration

The intensity and duration of exercise can also influence appetite regulation in obese individuals. Indeed, a study by Deighton et al. [110] found that high-intensity exercise led to greater appetite suppression than moderate-intensity exercise in overweight and obese individuals. Additionally, King et al. [111] demonstrated that appetite suppression was more pronounced following longer durations of exercise than shorter bouts.

Specifically, exercise can significantly regulate appetite in obese individuals by impacting hunger and satiety hormones, increasing energy expenditure, and modulating the effects of exercise intensity and duration. Incorporating regular physical activity into the treatment plan for obesity can help facilitate weight loss and long-term weight management by promoting healthier appetite regulation.

Physical activity is crucial in managing obesity and its associated health complications. Aerobic exercise, resistance training, combined aerobic and resistance exercise, and high-intensity interval training have all significantly improved CV and metabolic health, body composition, and overall quality of life in obese individuals.

Overall, the mechanisms through which diet and physical activity promote beneficial effects in obese patients include dietary modifications such as caloric restriction, with a reduced-calorie diet leading to a negative energy balance, forcing the body to utilize stored fat as an energy source, resulting in weight loss. Caloric restriction also enhances insulin sensitivity, reduces inflammation, and improves lipid profile, contributing to the overall improvement in metabolic health [112].

Specific macronutrient compositions, such as low-carbohydrate or low-fat diets, can elicit varying effects. Low-carbohydrate diets may promote greater weight loss and improved insulin sensitivity, while low-fat diets may reduce cardiovascular risk factors. Individualized dietary approaches are important, taking into consideration personal preferences, adherence, and metabolic characteristics [113].

Dietary fiber offers several benefits, including increased satiety, improved glycemic control, and reduced cardiovascular risk. High-fiber foods slow down gastric emptying, leading to prolonged feelings of fullness, which can help with appetite control and weight management. Micronutrient content with adequate intake of essential vitamins and minerals is crucial in supporting metabolic processes and overall health. Obese individuals often exhibit nutrient deficiencies, which can be addressed through a balanced diet or targeted supplementation, as advised by healthcare professionals [114,115].

On the other hand, engaging in physical activity increases energy expenditure, contributing to a negative energy balance and weight loss. Aerobic exercise, such as walking, jogging, or cycling, burn calories and promote cardiovascular fitness. Resistance training builds lean muscle mass, which increases resting metabolic rate and improves body composition [116].

Diet and physical activity can improve insulin sensitivity. Regular physical activity enhances insulin sensitivity, facilitating glucose uptake by muscles and reducing the risk of type 2 diabetes. Exercise stimulates glucose transporters on muscle cells, improving insulin-mediated glucose utilization and glycemic control [117].

Physical activity induces metabolic adaptations, including increased mitochondrial density, improved fatty acid oxidation, and enhanced lipid metabolism. These adaptations favor the utilization of stored fat as an energy source, leading to weight loss and improved metabolic health. Exercise can also influence appetite regulation by altering the production of appetite-related hormones, such as ghrelin and peptide YY. Acute exercise suppresses appetite, while chronic exercise training may lead to long-term appetite suppression and reduced calorie intake [118].

Dietary modifications and increased physical activity play crucial roles in managing obesity and improving overall health.

A personalized and comprehensive approach to exercise prescription should be implemented to address each individual’s unique needs and preferences, considering their goals, fitness levels, and potential barriers to adherence. Additionally, it is essential to promote a sustainable and enjoyable diet and exercise regimen that encourages long-term participation and fosters a healthier lifestyle. Ultimately, combining regular physical activity with a balanced and nutrient-dense diet will yield the most significant improvements in obesity management and overall health.

## 4. Contribution of Diet and Physical Activity in Improving Inflammatory Conditions in Obese Patients

Obesity is associated with a chronic state of low-grade inflammation, characterized by elevated levels of pro-inflammatory markers such as C-reactive protein (CRP), interleukin-6 (IL-6), and tumor necrosis factor-alpha (TNF-α). This systemic inflammation contributes to the development of various metabolic disorders and increases the risk of cardiovascular disease [119].

Dietary modifications can contribute to inflammatory management. Incorporating a diet rich in anti-inflammatory foods can help combat the inflammatory response in obese individuals. Foods such as fruits, vegetables, whole grains, fatty fish (rich in omega-3 fatty acids), nuts, and seeds are known for their anti-inflammatory properties [120]. These foods contain antioxidants, polyphenols, and other bioactive compounds that help reduce inflammation [121].

Omega-3 fatty acids, found in fatty fish, flaxseeds, chia seeds, and walnuts, have been shown to have anti-inflammatory effects [18]. They can suppress the production of pro-inflammatory cytokines and promote the synthesis of anti-inflammatory molecules, leading to a reduction in systemic inflammation [122].

Dietary fiber acts as a prebiotic, promoting the growth of beneficial gut bacteria. These bacteria produce short-chain fatty acids (SCFAs), such as butyrate, which have anti-inflammatory properties [22]. High-fiber foods, including fruits, vegetables, legumes, and whole grains, can help modulate the gut microbiota and reduce systemic inflammation [123].

The Mediterranean diet, rich in fruits, vegetables, whole grains, legumes, fish, and healthy fats, has been associated with a lower inflammatory profile. This dietary pattern provides a variety of anti-inflammatory nutrients and phytochemicals, contributing to the reduction of inflammation in obese individuals [124].

Physical activity can also greatly contribute to inflammatory management, as it has been shown to reduce adipose tissue inflammation, a key source of pro-inflammatory cytokines. Regular exercise promotes adipocyte browning, which increases the release of anti-inflammatory adipokines, such as adiponectin, while reducing the secretion of pro-inflammatory molecules [125].

Exercise can modulate the immune response, leading to an anti-inflammatory effect. It can reduce the production of pro-inflammatory cytokines and increase the release of anti-inflammatory cytokines [126]. This immune modulation helps attenuate the chronic low-grade inflammation associated with obesity [127].

Physical activity improves insulin sensitivity, reducing hyperinsulinemia, a condition that promotes inflammation. By enhancing glucose uptake and utilization in muscles, exercise helps maintain glycemic control and reduces the inflammatory response associated with insulin resistance [128].

Physical activity acts as a stress-reducing mechanism, which can indirectly affect inflammation. Chronic stress promotes the release of stress hormones, such as cortisol, which can contribute to systemic inflammation. Exercise helps mitigate the impact of stress on inflammation by reducing cortisol levels.

Dietary modifications and physical activity play significant roles in improving the inflammatory condition of obese patients. Anti-inflammatory foods, omega-3 fatty acids, fiber intake, and adherence to a Mediterranean-style diet contribute to reduced systemic inflammation. Physical activity reduces adipose tissue inflammation, modulates the immune response, improves insulin sensitivity, and aids in stress reduction. These interventions, when combined, offer a comprehensive approach to managing obesity-related inflammation. However, personalized recommendations and professional guidance are crucial for optimizing outcomes.

## 5. Pharmacological Treatments for Weight Reduction

Despite the increasing prevalence of obesity, effective and sustainable weight management solutions remain elusive. In this part of the review, we will provide an in-depth analysis of the available pharmacological treatments for weight reduction, focusing on their mechanisms of action, efficacy, and safety profiles (Table 1).

Specifically, pharmacological agents used for weight reduction can be categorized based on their mechanisms of action, which include appetite suppression, fat absorption inhibition, and increased energy expenditure [150].

### 5.1. Appetite Suppression

Central-acting agents, such as phentermine, lorcaserin, and the combination of bupropion–naltrexone, suppress appetite by modulating neurotransmitter levels in the brain. Phentermine, a sympathomimetic amine, increases the release of norepinephrine and dopamine, reducing hunger [151]. Lorcaserin, a selective serotonin 2C receptor agonist, enhances satiety signals by activating pro-opiomelanocortin neurons in the hypothalamus [148]. On the other hand, the combination of bupropion–naltrexone, by acting on both the dopaminergic and opioid systems, reduces food cravings and increases satiety [137]. Basically, pharmacological agents that induce appetite suppression aim to reduce calorie intake by modulating the complex neuroendocrine pathways responsible for hunger and satiety. Specifically, sibutramine and lorcaserin have been withdrawn from the market.

#### 5.1.1. Phentermine

Phentermine is a sympathomimetic amine that primarily acts as a norepinephrine-releasing agent. It stimulates the release of norepinephrine in the hypothalamus, increasing sympathetic nervous system activation and reducing appetite. The exact mechanisms underlying phentermine’s appetite-suppressing effects are not fully understood, but they are believed to involve modulation of the central nervous system’s noradrenergic and dopaminergic pathways [152]. Therefore, it is usually prescribed for short-term use (up to 12 weeks) in combination with lifestyle interventions, such as a low-calorie diet and increased physical activity.

Several clinical trials have demonstrated the efficacy of phentermine in promoting weight loss. A meta-analysis by Kang et al. (2016) [153] found that phentermine monotherapy resulted in a mean weight loss of 3.6 kg compared to placebo. In a 28-week randomized controlled trial (RCT), the phentermine 15 mg (half of the maximum approved dose) group (*n* = 108) achieved an average weight loss of 6.1% compared to 1.7% in the placebo group (*n* = 109) [154]. Additionally, phentermine is effective when combined with other weight-loss medications, such as topiramate [140].

Phentermine is generally well tolerated, with the most commonly reported side effects being dry mouth, insomnia, and constipation [151]. However, concerns have been raised regarding the potential CV risks associated with phentermine use, particularly in patients with pre-existing heart conditions [155]. Therefore, the FDA has recommended that phentermine be used for no more than 12 weeks, and it is contraindicated in patients with CV disease, uncontrolled hypertension, or a history of drug abuse (FDA, 2012).

Current literature supports phentermine as a short-term pharmacological therapy for obesity. Its appetite-suppressing effects contribute to significant weight loss in clinical trials. However, further research is needed to evaluate phentermine’s long-term safety and efficacy in obesity management.

#### 5.1.2. Sibutramine (Withdrawn from the Market)

Several clinical trials have demonstrated the effectiveness of sibutramine in promoting weight loss in obese patients. For example, a meta-analysis by Padwal and Majumdar (2007) reported a mean weight loss of 4.6 kg over 6–12 months when comparing sibutramine to a placebo [147]. Furthermore, this weight loss was accompanied by improvements in waist circumference, blood pressure, and lipid profiles.

However, it is essential to note that these benefits were only observed when sibutramine was used with a reduced-calorie diet and increased physical activity. Consequently, long-term adherence to lifestyle modifications is essential for maintaining weight loss, regardless of the pharmacological intervention [156].

Sibutramine has been associated with several adverse effects, including increased heart rate, elevated blood pressure, dry mouth, constipation, and insomnia [145]; these side effects often lead to discontinuation of the medication, limiting its long-term effectiveness.

More concerning are the potential CV risks associated with sibutramine use. Specifically, the Sibutramine Cardiovascular OUTcomes (SCOUT) trial, a large, multicenter, randomized, placebo-controlled study, showed a significantly amplified risk of non-fatal myocardial infarction and non-fatal stroke in patients treated with sibutramine [146]. In response to these findings, sibutramine was withdrawn from the market in many countries, including the United States and the European Union, in 2010.

While sibutramine has demonstrated efficacy in promoting weight loss in obese patients, its safety profile raises significant concerns: the potential CV risks associated with its use outweigh the benefits for most patients, leading to its withdrawal from the market in various countries.

#### 5.1.3. Lorcaserin (Withdrawn from the Market)

Lorcaserin is a selective serotonin 2C (5-HT2C) receptor agonist that acts primarily as an appetite suppressant. By selectively activating 5-HT2C receptors in the hypothalamus, lorcaserin promotes the release of proopiomelanocortin (POMC) neurons, suppressing appetite and increasing satiety [149]. This mechanism of action is distinct from earlier non-selective serotonin agonists, which were associated with significant CV risks due to their activation of 5-HT2B receptors [157].

Several clinical trials have demonstrated the efficacy of lorcaserin in promoting weight loss. The BLOOM (Behavioral Modification and Lorcaserin for Overweight and Obesity Management) trial, a 52-week, double-blind, placebo-controlled study, reported that participants receiving lorcaserin (10 mg twice daily) achieved a mean weight loss of 5.8%, compared to 2.2% in the placebo group [148]. The BLOSSOM (Behavioral Modification and Lorcaserin Second Study for Obesity Management) trial, another 52-week study, demonstrated a mean weight loss of 5.0% with lorcaserin, compared to 1.3% with placebo [158]. Similar results were observed in the BLOOM-DM (Behavioral Modification and Lorcaserin for Obesity and Overweight Management in Diabetes Mellitus) trial, which focused on participants with type 2 diabetes [159].

Lorcaserin has been generally well tolerated in clinical trials, with the most commonly reported side effects being headache, dizziness, and nausea [148]. Significantly, lorcaserin has not been associated with the CV risks observed with earlier non-selective serotonin agonists, likely due to its selective action on 5-HT2C receptors [160]. However, the FDA initially required post-marketing studies to further evaluate the CV safety of lorcaserin, and in 2020, it was withdrawn from the market due to concerns about an increased risk of cancer (FDA, 2020).

Before its withdrawal from the market, lorcaserin demonstrated promise as a pharmacological therapy for obesity, with its appetite-suppressing effects contributing to significant weight loss in clinical trials. While generally well tolerated, concerns about an increased cancer risk led to its withdrawal from the market. Further research is needed to evaluate lorcaserin’s long-term safety and efficacy and other selective serotonin agonists in obesity management.

#### 5.1.4. Bupropion–Naltrexone

Bupropion–naltrexone is thought to act synergistically to promote weight loss by targeting both the reward and control centers in the brain. Bupropion increases the levels of dopamine and norepinephrine, which are associated with appetite regulation and energy expenditure; at the same time, naltrexone blocks opioid receptors, reducing the rewarding effects of food and enhancing the action of bupropion [137].

Several clinical trials have demonstrated the efficacy of bupropion–naltrexone in promoting weight loss. The COR-I (Contrave Obesity Research I) trial, a 56-week, double-blind, placebo-controlled study, reported that participants receiving bupropion–naltrexone (32 mg naltrexone/360 mg bupropion daily) achieved a mean weight loss of 6.1%, compared to 1.3% in the placebo group [137]. Similar results were observed in the COR-II trial, with a mean weight loss of 6.4% in the bupropion–naltrexone group compared to 1.2% in the placebo group [136].

Bupropion–naltrexone has been generally well tolerated in clinical trials, with the most commonly reported side effects being nausea, constipation, headache, and dizziness [137]. However, concerns have been raised regarding the potential CV risks associated with bupropion use, particularly in patients with pre-existing heart conditions [135]. Therefore, the FDA has recommended that bupropion–naltrexone be used cautiously in patients with a history of CVD, uncontrolled hypertension, or seizure disorders (FDA, 2014).

Current literature supports the use of bupropion–naltrexone as a pharmacological therapy for obesity. Its synergistic action on appetite regulation and reward pathways contributes to significant weight loss in clinical trials. However, further research is needed to evaluate bupropion–naltrexone’s long-term safety and efficacy in obesity management.

### 5.2. Fat Absorption Inhibition—Orilstat

Orlistat works by inhibiting pancreatic and gastric lipases, breaking down dietary fats into absorbable free fatty acids. By reducing the absorption of dietary fat, orlistat leads to a decrease in caloric intake and subsequent weight loss.

Several clinical trials have demonstrated the efficacy of orlistat in promoting weight loss. A meta-analysis by Rucker et al. [161] found that orlistat treatment resulted in a mean weight loss of 2.89 kg compared to placebo. In a 12-week, double-blind, placebo-controlled trial, Davidson et al. [131] reported that participants receiving orlistat (120 mg three times daily) achieved a mean weight loss of 4.3 kg, compared to 2.8 kg in the placebo group. Orlistat has also been shown to be effective in maintaining weight loss over the long term [130].

Orlistat has been generally well tolerated in clinical trials, with the most commonly reported side effects being gastrointestinal, such as oily spotting, fecal urgency, and flatulence. However, concerns have been raised regarding the potential for malabsorption of fat-soluble vitamins and the risk of liver injury [129]. Therefore, the FDA has recommended that orlistat be used with caution in patients with a history of cholestasis or malabsorption syndrome (FDA, 2010).

Current literature supports the use of orlistat as a pharmacological therapy for obesity. Its inhibition of pancreatic and gastric lipases contributes to significant weight loss in clinical trials. While generally it is well tolerated, concerns regarding the potential for malabsorption of fat-soluble vitamins and the risk of liver injury warrant caution in prescribing orlistat, particularly for patients with pre-existing liver or gastrointestinal conditions. Nevertheless, further research is needed to evaluate the long-term safety and efficacy of orlistat in obesity management.

### 5.3. Increased Energy Expenditure

Pharmacological agents that increase energy expenditure promote weight loss by boosting the body’s metabolic rate, leading to higher energy consumption and increased calorie burning. These agents typically target thermogenesis or mitochondrial function to enhance energy expenditure. Examples include thermogenic supplements, thyroid hormone analogs, and agents that modulate brown adipose tissue (BAT) activity.

#### 5.3.1. Thermogenic Supplements

Caffeine and capsaicin are thermogenic supplements that increase energy expenditure by enhancing thermogenesis. Thermogenesis is the generation of heat within the body, contributing to calorie burning. Caffeine, a natural stimulant in coffee and tea, boosts the metabolic rate by increasing catecholamine release (e.g., epinephrine and norepinephrine), stimulating fatty acid breakdown, and raising energy expenditure. Likewise, capsaicin, the active compound in chili peppers, increases energy expenditure by activating transient receptor potential vanilloid 1 (TRPV1) channels, enhancing thermogenesis and fat oxidation.

#### 5.3.2. Thyroid Hormone Analogs

Thyroid hormones play a crucial role in regulating energy metabolism, as they modulate the basal metabolic rate and the activity of enzymes involved in energy expenditure. Thyroid hormone analogs, such as 3,5-diiodo-L-thyronine (T2) and 3,3’,5-triiodo-L-thyronine (T3), have been investigated for their potential to increase energy expenditure and promote weight loss [162]. These analogs stimulate mitochondrial biogenesis and activity, increasing energy consumption and weight loss. However, their use has been limited due to potential adverse effects on the CV system and bone metabolism.

#### 5.3.3. Modulation of Brown Adipose Tissue (BAT) Activity

BAT is a specialized fat that generates heat through non-shivering thermogenesis. This process is primarily regulated by uncoupling protein 1 (UCP1), expressed in the inner mitochondrial membrane of brown adipocytes. Activation of UCP1 uncouples oxidative phosphorylation from ATP production, leading to increased energy expenditure in heat [163]. Several pharmacological agents, such as β3-adrenergic receptor agonists (e.g., mirabegron) and fibroblast growth factor 21 (FGF21) analogs, have been investigated for their potential to activate BAT and increase energy expenditure [164,165]. However, further research is needed to establish their safety and efficacy in promoting weight loss.

In conclusion, pharmacological agents that increase energy expenditure offer a promising approach to weight loss by enhancing metabolic rate, thermogenesis, or BAT activity. Although some agents have shown potential in preclinical and clinical studies, further research is needed to establish their safety, efficacy, and long-term effects on weight management.

### 5.4. Combination Therapies and Future Directions

#### 5.4.1. Phentermine–Topiramate

In recent clinical trials, the combination of phentermine, a sympathomimetic amine, and topiramate, an antiepileptic drug, has shown promise as an anti-obesity agent [140].

Phentermine–topiramate is thought to synergistically promote weight loss by targeting appetite regulation and energy expenditure. Phentermine stimulates the release of norepinephrine, which suppresses appetite; at the same time, topiramate is believed to enhance the action of gamma-aminobutyric acid (GABA) and modulate voltage-gated ion channels, leading to appetite suppression and increased satiety [139].

Several clinical trials have demonstrated the efficacy of phentermine–topiramate in promoting weight loss. The CONQUER (Controlled-Release Phentermine/Topiramate in Obese Adults) trial, a 56-week, double-blind, placebo-controlled study, reported that participants receiving phentermine–topiramate achieved a mean weight loss of 9.8%, compared to 1.2% in the placebo group [140]. Similar results were observed in the EQUIP (Effect of Qsymia on Weight Loss in Obese Subjects) trial, with a mean weight loss of 10.9% in the phentermine–topiramate group compared to 1.6% in the placebo group [138].

Specifically, phentermine–topiramate has been generally well tolerated in clinical trials, with the most commonly reported side effects being dry mouth, constipation, and paresthesia [140]. However, concerns have been raised regarding the potential CV risks associated with phentermine use and the risk of birth defects associated with topiramate use (FDA, 2012). Therefore, the FDA has recommended that phentermine–topiramate be used with caution in patients with a history of CVD, glaucoma, or hyperthyroidism and that it should not be used during pregnancy (FDA, 2012).

Current literature supports the use of phentermine–topiramate as a pharmacological therapy for obesity. Moreover, its synergistic action on appetite regulation and energy expenditure contributes to significant weight loss in clinical trials. Finally, further research is needed to evaluate phentermine–topiramate’s long-term safety and efficacy in obesity management.

#### 5.4.2. GLP-1 Receptor Agonists

GLP-1 receptor agonists, initially developed and approved for treating type 2 diabetes, have demonstrated promising results in promoting weight loss and improving metabolic parameters in individuals with obesity. These agents, including liraglutide and semaglutide, mimic the action of endogenous GLP-1, a hormone involved in glucose homeostasis and appetite regulation. By activating GLP-1 receptors, these drugs exert their effects through multiple mechanisms, including increased insulin secretion, reduced glucagon release, delayed gastric emptying, and modulation of appetite-regulating brain regions. Consequently, GLP-1 receptor agonists have been shown to promote satiety, reduce food intake, and facilitate weight loss in clinical trials [166,167,168].

#### 5.4.3. Liraglutide

Initially approved for the treatment of type 2 diabetes, it has shown promise as an anti-obesity agent in recent clinical trials [134].

Liraglutide is a GLP-1 receptor agonist that acts primarily by mimicking the action of endogenous GLP-1, a hormone that regulates glucose homeostasis and appetite. Liraglutide promotes satiety and reduces food intake by slowing gastric emptying and increasing insulin release in response to elevated blood glucose levels [133].

Several clinical trials have demonstrated the efficacy of liraglutide in promoting weight loss. The SCALE (Satiety and Clinical Adiposity—Liraglutide Evidence) Obesity and Prediabetes trial reported that participants receiving liraglutide achieved a mean weight loss of 8.4%, compared to 2.8% in the placebo group [132]. Moreover, in a 20-week, double-blind, placebo-controlled trial, Astrup et al. [134] found that participants receiving liraglutide achieved a mean weight loss of 7.2 kg, compared to 2.8 kg in the placebo group.

Liraglutide has been generally well tolerated in clinical trials, with the most commonly reported side effects being gastrointestinal, such as nausea, vomiting, and diarrhea [132]. However, concerns have been raised regarding the potential risk of pancreatitis and thyroid C-cell tumors associated with liraglutide use (FDA, 2014). Consequently, the FDA has recommended that liraglutide be used with caution in patients with a history of pancreatitis or medullary thyroid carcinoma (FDA, 2014).

Current literature supports liraglutide as a pharmacological therapy for obesity; however, further research is needed to evaluate the long-term safety and efficacy of liraglutide in obesity management.

#### 5.4.4. Semaglutide

Semaglutide is a GLP-1 receptor agonist which mimics the action of endogenous GLP-1, a hormone involved in glucose homeostasis and appetite regulation [144]. The STEP (Semaglutide Treatment Effect in People with obesity) clinical trial program has provided substantial evidence for the efficacy of semaglutide in obesity management. In STEP 1, participants receiving semaglutide achieved a mean weight loss of 14.9%, compared to 2.4% in the placebo group [168]. STEP 2, which focused on participants with type 2 diabetes, demonstrated a mean weight loss of 9.6% with semaglutide, compared to 3.4% with placebo [143]. Similar results were observed in STEP 3 and STEP 4, further supporting the efficacy of semaglutide in promoting weight loss [141,142].

Semaglutide has been generally well tolerated in clinical trials, with gastrointestinal side effects being the most commonly reported adverse events [168]. These side effects, including nausea, vomiting, and diarrhea, were mainly mild to moderate in severity and tended to decrease over time [143]. Serious adverse events were infrequent and comparable between semaglutide and placebo groups [141].

Current literature supports the use of semaglutide as a promising pharmacological therapy for obesity. While gastrointestinal side effects are common, they are generally mild and decrease over time; however, further research is needed to evaluate the long-term safety and efficacy of semaglutide in obesity management.

### 5.5. Future Directions

With obesity rates continuing to rise, the development of novel pharmacological treatments remains a priority. Research is currently focused on understanding the complex neuroendocrine pathways regulating energy homeostasis and appetite control. Emerging targets include the melanocortin-4 receptor (MC4R), neuropeptide Y, and ghrelin [169]. Moreover, investigating the potential of gene therapy, stem cell therapy, and personalized medicine approaches may also yield innovative weight management solutions in the future.

## 6. Surgical Treatments

While lifestyle changes such as diet and exercise can help with weight loss, some people may require more intensive treatment options such as obesity surgical treatments.

Obesity surgical treatments, or bariatric surgery, are procedures designed to help people lose weight by reducing the stomach size or bypassing part of the small intestine. There are three main types of bariatric surgery: restrictive procedures (laparoscopic adjustable gastric band (LAGB), vertical banded gastroplasty (VBG)), malabsorptive procedures (biliopancreatic diversion, biliopancreatic diversion with duodenal switch), and combined procedures (Roux-en-Y gastric bypass (RYGBP), Long-limb RYGBP) [170]. According to the guidelines, all individuals with a BMI > 35 kg/m^2^ may be considered for surgery, irrespective of the presence of underlying health problems, while those with a BMI > 30 Kg/m^2^ may be recommended for surgery in cases of diabetes or for those who have not been able to maintain long-lasting weight loss. It is important to note that BMI thresholds should be adjusted for the Asian population, since individuals in those communities typically experience adverse health outcomes at a lower BMI [171].

Many studies have compared the efficacy of different surgical techniques on weight loss; more precisely, they showed that malabsorptive procedures could obtain a major weight loss than restrictive procedures [172]. They have also demonstrated that obesity surgical treatments can significantly impact CV risk factors. The Swedish Obese Subjects (SOS) study is the first long-term, prospective, controlled trial to provide information on bariatric surgery’s effects on CVD incidence [173]. Compared with medical care, bariatric surgery was associated with a long-term reduction in overall mortality and decreased incidences of diabetes, myocardial infarction, stroke, and cancer.

The 10-year follow-up results of the SOS study showed that the surgical group had higher rates of normalization of blood glucose values, triglyceridemia, cholesterol-HDL, and blood pressure [173]. In the surgical group, largely independent of the type of surgery performed, there was a marked reduction in the incidence of new cases of diabetes, with a relative risk respected against the control group of 0.12 (95% CI 0.05–0.27; *p* < 0.001) for gastric bypass, 0.20 (95% CI 0.13–0.32; *p* < 0.001) for gastric banding, and 0.25 (95% CI 0.19–0.31; *p* < 0.001) for vertical gastroplasty [173].

A study published in the American Journal of Hypertension found that patients who underwent gastric bypass surgery significantly reduced their blood pressure compared to those who did not. Moreover, surgery-induced body weight loss in morbidly obese hypertensive subjects with impaired circadian BP variation is not only associated with BP reduction but also with the restoration of normal BP rhythm [174].

The GATEWAY Randomized Trial (Gastric Bypass to Treat Obese Patients With Steady Hypertension) included patients with hypertension and obesity (BMI between 30 and 39.8 kg/mq) and compared the outcome between the patients that were undergoing Roux-en-Y gastric bypass plus medical therapy or medical therapy alone [175]. Results highlighted a remission of hypertension in 51% of patients randomized to gastric bypass considering office and 24-h ambulatory blood pressure monitoring, respectively, whereas no patient submitted to medical therapy was free of antihypertensive drugs at 12 months [175].

Another study published in the Journal of the American Medical Association confirmed that patients who underwent bariatric surgery (in this case, gastric bypass surgery) had a 40% reduction in their risk of developing CVD compared to those who did not have surgery [176]. In addition to reducing CV risk factors, obesity surgical treatments can also improve CV function. A study published in the Journal of the American College of Cardiology found that patients who underwent gastric bypass had improved left ventricular function, an essential measure of heart health [177].

One of the ways that obesity surgical treatments can reduce CV risk is also by improving insulin sensitivity. Indeed, insulin resistance is a common problem in people who are obese, and it is a significant risk factor for CVD. Obesity surgical treatments can improve insulin sensitivity by reducing the amount of fat in the body and improving glucose metabolism. A randomized trial compared Roux-en-Y Gastric Bypass or sleeve gastrectomy versus Intensive Medical Management for the Control of Type 2 Diabetes [178]. The use of drugs to lower glucose, lipid, and blood-pressure levels decreased significantly after both surgical procedures but increased in patients receiving medical therapy only [178].

While obesity surgical treatments can significantly benefit CV health, they are not without risks. Complications can include bleeding, infection, and thrombosis; consequently, it is important for patients to discuss the risks and benefits of obesity surgical treatments with their healthcare provider before making a decision [179].

In conclusion, obesity surgical treatments can significantly impact CV risk factors and improve CV function. While they are not without risks, they can be an effective treatment option for people who are struggling with obesity and its associated health problems. Considering obesity, surgical treatments require an evaluation of risks versus benefits by the healthcare provider.

## 7. How to Select the Most Appropriate Intervention

Multiple therapeutic modalities, including lifestyle interventions, behavioral therapy, pharmacotherapy, and bariatric surgery, can be used in the treatment of obesity [180]; more precisely, the selection of the most appropriate weight-loss intervention will usually depend on the degree of excess weight of the patient and comorbidities [65,181,182].

Accordingly, the Edmonton obesity staging system (EOSS) has been used to provide additional guidance for therapeutic interventions in individual patients [183]. Hypothetically, EOSS stages 0 and 1 should be managed in a community and primary care setting, while EOSS stages 3 and 4 should be dealt with through pharmacological and surgical management in specialist centers.

## 8. Artificial Intelligence in Obesity Management

Artificial Intelligence (AI) is the technological acquisition of knowledge and skill by a nonhuman device that, after being programmed initially, performs adaptive output tasks based on data input learnings [184]. More precisely, AI has several areas of opportunity in CV prevention and the battle against chronic diseases such as obesity [19,32,103]. Firstly, AI may help doctors who specialize in obesity by offering interactive programming related to analyses of body composition imaging for more accurate valuation of anthropometrics, behavior coaching, personal nutritional intervention, and physical activity recommendations, predictive modeling for identifying patients at risk for obesity-related complications, and implementation of precision/personalized medicine [184,185,186,187,188]. Moreover, AI has multiple medical education applications, via personalized learning, virtual reality, and intelligent tutoring systems [184]. AI may augment telemedicine, scheduling appointments and integrating remote monitoring of patients (e.g., mobile applications and wearable technologies) [189]. Finally, this new approach may help identify patterns in datasets related to a medical practice or institution (i.e., analytics related to electronic health records), such as revealing areas where potential improvements in health outcomes might be achieved with changes in clinician management [184,190,191].

AI is contributing to an evolution in medical care, including managing obese patients. However, challenges of AI still include ethical and legal concerns (e.g., privacy and confidentiality), accuracy and reliability, and potential perpetuation of pervasive systemic biases.

## 9. Conclusions and Future Directions

Obesity is a heterogeneous condition in which CVD risk profiles are not mediated solely by overall body fat mass but depend mainly on individual differences in regional body fat distribution, which negatively involve cardiac structure and function. With increasing prevalence of obesity in populations, there is a need for interventions for primary prevention and better treatment of obesity as a chronic disease. More precisely, a comprehensive assessment and multiple interventions are needed to reduce CVD risk and mortality. Moreover, innovative and alternative interventions should be considered through AI to support all interventions and ensure high adherence to the therapy over time, to improve quality of life, healthy behaviors, and CV outcome.

## Figures and Tables

**Table 1:** Pharmacological treatments for weight reduction.

Drug Name	Drug Class	Mechanism of Action	Indications	Common Side Effects
**Orlistat (Xenical) [129,130,131]**	Lipase inhibitor	Inhibits gastric and pancreatic lipases, reducing fat absorption in the gut	Obesity weight management	Gastrointestinal issues, oily stools, flatulence
**Liraglutide (Saxenda) [132,133,134]**	GLP-1 receptor agonist	Mimics the action of incretin hormones, increasing satiety and reducing appetite	Obesity weight management type 2 diabetes	Nausea, vomiting, diarrhea, constipation
**Naltrexone–Bupropion (Contrave) [135,136,137]**	Opioid antagonist and dopamine/norepinephrine reuptake inhibitor	Reduces appetite by blocking opioid receptors and increasing dopamine/norepinephrine levels	Obesity weight management	Nausea, constipation, headache, dizziness, dry mouth
**Phentermine-Topiramate (Qsymia) [138,139,140]**	Sympathomimetic amine and anticonvulsant	Increases satiety and reduces appetite through increased norepinephrine release and GABA receptor modulation	Obesity weight management	Dizziness, dry mouth, constipation, insomnia, palpitations
**Semaglutide (Wegovy) [141,142,143,144]**	GLP-1 receptor agonist	Mimics the action of incretin hormones, increasing satiety and reducing appetite	Obesity weight management type 2 diabetes	Nausea, vomiting, diarrhea, constipation, abdominal pain
**Sibutramine (Meridia) [145,146,147]**	Serotonin-norepinephrine reuptake inhibitor	Increases satiety and reduces appetite by enhancing serotonin and norepinephrine release	Obesity weight management	Dry mouth, constipation, insomnia, increased heart rate
**Lorcaserin (Belviq) [148,149]**	Serotonin 2C receptor agonist	Activates serotonin 2C receptors in the brain to promote satiety and reduce appetite	Obesity weight management	Headache, dizziness, fatigue, nausea, dry mouth, constipation
**Thermogenic Supplements**	Dietary supplements	Increase metabolism and promote fat burning by increasing thermogenesis	Weight management fat loss	Varies depending on ingredients: insomnia, increased heart rate, anxiety, gastrointestinal discomfort

## Data Availability

No new data were created or analyzed in this study. Data sharing is not applicable to this article.
